# Inhibition does not affect the timing code for vocalizations in the mouse auditory midbrain

**DOI:** 10.3389/fphys.2014.00140

**Published:** 2014-04-16

**Authors:** Alexander G. Dimitrov, Graham I. Cummins, Zachary M. Mayko, Christine V. Portfors

**Affiliations:** ^1^Department of Mathematics, Washington State University VancouverVancouver, WA, USA; ^2^School of Biological Sciences, Washington State University VancouverVancouver, WA, USA

**Keywords:** spike timing, mouse IC, inhibition, selectivity, information, neural coding

## Abstract

Many animals use a diverse repertoire of complex acoustic signals to convey different types of information to other animals. The information in each vocalization therefore must be coded by neurons in the auditory system. One way in which the auditory system may discriminate among different vocalizations is by having highly selective neurons, where only one or two different vocalizations evoke a strong response from a single neuron. Another strategy is to have specific spike timing patterns for particular vocalizations such that each neural response can be matched to a specific vocalization. Both of these strategies seem to occur in the auditory midbrain of mice. The neural mechanisms underlying rate and time coding are unclear, however, it is likely that inhibition plays a role. Here, we examined whether inhibition is involved in shaping neural selectivity to vocalizations via rate and/or time coding in the mouse inferior colliculus (IC). We examined extracellular single unit responses to vocalizations before and after iontophoretically blocking GABA_A_ and glycine receptors in the IC of awake mice. We then applied a number of neurometrics to examine the rate and timing information of individual neurons. We initially evaluated the neuronal responses using inspection of the raster plots, spike-counting measures of response rate and stimulus preference, and a measure of maximum available stimulus-response mutual information. Subsequently, we used two different event sequence distance measures, one based on vector space embedding, and one derived from the Victor/Purpura *D*_*q*_ metric, to direct hierarchical clustering of responses. In general, we found that the most salient feature of pharmacologically blocking inhibitory receptors in the IC was the lack of major effects on the functional properties of IC neurons. Blocking inhibition did increase response rate to vocalizations, as expected. However, it did not significantly affect spike timing, or stimulus selectivity of the studied neurons. We observed two main effects when inhibition was locally blocked: (1) Highly selective neurons maintained their selectivity and the information about the stimuli did not change, but response rate increased slightly. (2) Neurons that responded to multiple vocalizations in the control condition, also responded to the same stimuli in the test condition, with similar timing and pattern, but with a greater number of spikes. For some neurons the information rate generally increased, but the information per spike decreased. In many of these neurons, vocalizations that generated no responses in the control condition generated some response in the test condition. Overall, we found that inhibition in the IC does not play a substantial role in *creating the distinguishable and reliable neuronal temporal spike patterns* in response to different vocalizations.

## 1. Introduction

Many animals, including humans, use a wide variety of acoustically complex sounds to convey different types of information to members of their own species. For example, vocalizations used by male Mexican free-tailed bats when courting a female are acoustically different than those used to defend a territory (Bohn et al., 2009). For appropriate communication to occur, the receiving animal must reliably recognize and discriminate among different vocalizations. Therefore, the information in each vocalization must be coded by neurons in the auditory system. The responses of individual neurons in the auditory system can be used to discriminate among different vocalizations often because different firing rates are evoked by different vocalizations (Klug et al., 2002; Suta et al., 2003; Schneider and Woolley, 2010; Huetz et al., 2011; Mayko et al., 2012; Gaucher et al., 2013). However, in some neurons, vocalizations are poorly discriminated based on firing rate alone (Suta et al., 2003; Schneider and Woolley, 2010; Huetz et al., 2011; Woolley and Portfors, 2013). There is growing evidence that for some neurons, discrimination ability improves when measures of spike timing are included (Huetz et al., 2011; Woolley and Portfors, 2013). Thus, the auditory system may utilize two strategies for discriminating among vocalizations. The first is to create highly selective neurons such that only a small number of specific vocalizations evoke a strong response from each neuron, and the second is to create different spike timing patterns for different vocalizations such that each neural response can be matched to a specific vocalization.

Neural selectivity to vocalizations based on response rate occurs at multiple levels of the auditory system but the best evidence for where this selectivity is created comes from studies in the inferior colliculus (IC) (Klug et al., 2002; Xie et al., 2005; Mayko et al., 2012). The IC is the major processing and integrating center in the auditory midbrain (Winer and Schreiner, 2005) as it receives massive ascending projections from all auditory brainstem nuclei (Adams, 1979; Brunso-Bechtold et al., 1981; Frisina et al., 1998) as well as descending projections from the auditory thalamus and cortex (Saldana et al., 1996; Winer et al., 1998). Along with glutamatergic, GABAergic, and glycineric projections (Willard and Ryugo, 1983; Saint Marie and Baker, 1990; Saint Marie, 1996; Cant, 2005; Schofield, 2005), the IC receives a variety of modulatory inputs including those that are serotonergic (Hurley and Pollak, 2005) and dopaminergic (Tong et al., 2005). This convergence of inputs onto single neurons in the IC plays a fundamental role in shaping response properties to complex sounds, and creating selectivity to vocalizations. In particular, pharmacologically blocking GABAergic and glycinergic receptors in the IC decreases selectivity to social vocalizations in both bats (Klug et al., 2002; Xie et al., 2005) and mice (Mayko et al., 2012). In contrast, blocking inhibition in the nuclei of the lateral lemniscus does not alter neural selectivity to social vocalizations (Xie et al., 2005), suggesting that inhibition reduces the number of vocalizations that evoke responses from individual neurons in the IC. In these studies, selectivity was calculated based only on response rate, even though the spiking patterns of individual neurons to different vocalizations were often visibly distinct and sometimes altered when inhibition was blocked (Mayko et al., 2012). Thus, different temporal spiking patterns in IC neurons may be a coding strategy for discriminating among vocalizations, and the balance between excitation and inhibition may underlie these different temporal spiking patterns.

There is evidence in both the IC of mice and the MLd of zebra finches that temporal spiking patterns can code different vocalizations (Woolley and Portfors, 2013). In the IC of mice, spike timing information provides greater mutual information in responses to vocalizations than response rate for some neurons in IC, and in the MLd of finches discrimination among different songs is better using spike timing than spike rate for some neurons (Schneider and Woolley, 2010). Similarly, in the auditory cortex, temporal spike patterns provide a coding strategy for discriminating among vocalizations (Narayan et al., 2006; Schnupp et al., 2006; Wang et al., 2007; Recanzone, 2008; Huetz et al., 2009).

Only one study has assessed how the balance of excitation and inhibition may create highly reliable and different temporal spike patterns to different vocalizations (Gaucher et al., 2013). In this study, blocking inhibition in the auditory cortex increased the response rate, the reliability of temporal spiking patterns, and the amount of mutual information conveyed by individual recording sites but did not alter the amount of information conveyed by the population of cortical neurons. These results suggest that intracortical inhibition plays a role in reducing redundancy between cortical sites and thus leads to more efficient encoding of vocalizations.

How inhibition affects the timing code for vocalizations in the IC is not known. To address this question, we applied a variety of neurometrics to vocalization-evoked neuronal responses that were obtained before and after pharmacologically blocking GABAergic and glycinergic receptors. We found that in most neurons, blocking inhibition increased response rate and increased total mutual information. However, the information per spike was reduced. In addition, spike timing was generally unaffected by altering the balance between excitation and inhibition in the IC. This suggests that inhibition can increase selectivity to vocalizations by altering the excitability of neurons (iceberg effect) (Creutzfeldt et al., 1974), but in general it does not play a substantial role in creating the distinguishable and reliable temporal spike patterns in response to different vocalizations.

## 2. Methods

### 2.1. Experimental procedures

We recorded auditory responses from single neurons in the IC of awake, restrained CBA/CaJ mice. All mice were female less than 1 year old. Animals were housed with same-sex litter mates on a reversed 12 h light/dark schedule. All mice had *ad libitum* access to food and water. All animal care and experimental procedures were in accordance with the guidelines of the National Institutes of Health, and were approved by the Washington State University Institutional Animal Care and Use Committee.

#### 2.1.1. Surgical procedures

Surgical procedures were the same as in Mayko et al. (2012). Briefly, animals were anesthetized with isoflurane so that we could mount a headpost onto the skull with ultraviolet-cured dental cement (Muniak et al., 2012). We made a craniotomy (usually 1 mm × 1 mm) over top of the left inferior colliculus (IC), covered the hole with petroleum jelly or bone wax to prevent the brain from dehydrating, applied a local anesthetic (lidocaine) and an antibiotic (Neosporin) to the exposed muscle, and returned the mouse to its home cage to recover from surgery for at least 1 day before electrophysiological recordings.

#### 2.1.2. Acoustic stimulation

Acoustic stimulation was computer-controlled and included tone bursts (100 ms duration, 1 ms rise/fall time, 4 per second) and a suite of mouse vocalizations used in previous studies of mouse IC (Portfors et al., 2009; Mayko et al., 2012). All stimuli were stored in the computer and were output through a high speed, 16-bit digital-to-analog converter (Microstar Laboratories, Bellevue, WA, USA; 400,000 samples/s), fed to a programmable attenuator (Tucker Davis Technologies, Alachua, FL, USA; PA5), a power amplifier (Parasound), and to a leaf tweeter speaker (Emit) located 10 cm away from the mouse. We tested the acoustic properties of the system using a 1/4 inch calibrated microphone (Bruel and Kjaer, Denmark; model 4135) placed in the position normally occupied by the animal's ear. There was a smooth, gradual decrease in sound pressure from 6 to 100 kHz of about 3 dB per 10 kHz. Distortion components in tonal stimuli were buried in the noise floor, at least 50 dB below the signal level, as measured by custom-designed software performing a fast Fourier transform of the digitized microphone signal.

#### 2.1.3. Electrophysiological recording and drug application

We conducted electrophysiological experiments in a single-walled sound-attenuating chamber. On experimental days, we placed the animal securely into a foam body mold and attached the headpost to a custom-made stereotax apparatus (Muniak et al., 2012). If at any time during the experiment the animal showed signs of distress, the experiment was terminated. Experimental sessions lasted 4–5 h and we used each animal in 1–3 sessions.

The experimental procedures were the same as in Mayko et al. (2012). We obtained responses of single units to pure tones and mouse vocalizations before and after the application of the GABA_A_R and GlyR antagonists bicuculline and strychnine, respectively. The GABA_A_R antagonist bicuculline has also been shown to affect calcium-dependent potassium channels (Kurt et al., 2006), which are also present in the IC (Kelly and Caspary, 2005). We used a single micropipette electrode mounted on a five-barreled pipette for microiontophoretic application of drugs (Havey and Caspary, 1980). The tip of the single electrode extended 10–25 μm beyond the multibarrel pipette and contained 1 M NaCl. We broke the tip of the multibarrel pipette to a diameter of approximately 30 μm. We filled the center barrel of the multibarrel pipette with 1 M NaCl and connected it to a sum channel to balance all currents used to apply or retain drugs. The rest of the barrels contained the GABA_A_R antagonists bicuculline (10 mM, pH 3.0, vehicle 0.9% physiological saline; Sigma) and the GlyR antagonist strychnine (10 mM, pH 3.0, vehicle 0.9% physiological saline; Fluka, Milwaukee, WI). We used similar iontophoresis currents for drug retention and ejection to those used in previous studies (Wenstrup and Leroy, 2001; Ingham and McAlpine, 2005; Sanchez et al., 2008; Mayko et al., 2012). Bicuculline and strychnine were retained with negative current (−15 nA each) and ejected with positive current (range, +10 to +40 nA each).

We prepared all drugs and recording solutions the day of the experiment. We inserted separate silver wires into each barrel of the micropipette electrode and connected them to a microiontophoresis current generator (model 650, David Kopf Instruments, Tujunga, CA) to separately control the retention and ejection currents for each drug. We advanced the electrodes into the IC using a hydraulic micropositioner (David Kopf Instruments, Tujunga, CA) located outside the acoustic chamber. Extracellular action potentials were amplified (Dagan Corporation, Mineapolis, MN, USA), filtered (bandpass, 500–6000 Hz; Krohn-Hite, Brockton, MA, USA) and sent through a spike enhancer (Fredrick Haer, Bowdoin, ME, USA) before being digitized (Microstar Laboratories, Bellevue, WA, USA; 10,000 samples/s). Neural waveforms were displayed and archived using custom-written C++ software. Waveforms, raster plots, peri-stimulus time histograms (PSTHs), and statistics were viewed on-line and stored for off-line analysis.

We used tone bursts as search stimuli (varying duration, 1 ms rise/fall time) to obtain well isolated single units. We obtained characteristic frequency (CF) and minimum threshold (MT) of each single unit audiovisually. We defined CF as the frequency that evoked a response to 50% of the stimulus presentations at the lowest intensity, and MT as the lowest intensity that evoked a response 50% of the time to the CF. We obtained responses to vocalizations by presenting the suite of 14 vocalizations (variable duration, 1 ms rise/fall time, 4/s, 200-ms recording window) 10–40 times at multiple intensities. We then applied the GABA_A_R and GlyR antagonists and repeated presentation of the vocalization stimuli. We ejected bicuculline and strychnine together because we were interested in the general effects of inhibition on temporal responses to vocalizations rather than the separate effects of GABAergic and glycinergic inhibition. We initially applied low ejection currents (+10 nA) and then gradually increased the current if there was no effect. Once the response reached a steady-state, we kept the ejection currents at this level.

### 2.2. Analytical methods

#### 2.2.1. Data processing

Spike counts and raw waveforms were stored in the computer during data collection. We examined raw waveforms off-line to ensure only spikes from well isolated single units were used in the data analysis. Single units had signal-to-noise ratios of at least 4:1 and an inter-spike interval of at least 2 ms. The data collection software automatically detects the peak times of spikes to within the sampling precision of 2 μs. For analyses here, we used only the identified spike times.

Stimuli were typically presented at several different amplitude levels. Responses to different amplitudes of the same vocalization can appear quite different, so we treated each amplitude as a separate stimulus case. Analysis in this paper only includes a single amplitude for each stimulus, which is the one that produced the most response spikes in the control condition^1^.

The 14 vocalization stimuli had different durations, ranging from 10 to 143 ms. Analysis routines considered a time window within the responses, anchored to the stimulus presentation time. The recorded data consist of the time window [−20, 200], that is, starting 20 ms before the stimulus begins, and lasting 200 ms afterwards (which is 57 ms after the end of the longest stimulus). Presentation of raw data shows the time window [0, 200]. Analysis typically used a more restricted window. Unless otherwise stated, this window was [0, 158], that is, the start time of stimulus presentation until 15 ms after the end of the longest stimulus.

#### 2.2.2. Mutual information

Because the stimulus in each trial is 1 of 14 distinct waveforms, we describe the stimulus as an integer label between 0 and 13. The responses consist of event sequences in a window of time around the stimulus. In order to estimate a mutual information, we first mapped the responses onto a set of distinct response *classes* (Dimitrov and Miller, 2001), which are also represented with integer labels. We considered a range of methods for mapping responses to classes, and for subsequent estimates of mutual information between the sets of stimulus and response labels. What is presented here is the protocol that provided the best performance on artificial data sets, where we knew the true stimulus/response relationships, and could thus test the estimates.

The *inverse spike time* representation of a response is a vector of length *L*, where *L* is the maximum number of spikes that occurs in any of the responses being considered. This vector has components $r_{ist}=\{\frac{1}{t_{1}},\;\frac{1}{t_{2}}\cdots \;\frac{1}{t_{L}}\}$. The values *t*_*i*_ for a response containing *l* < *L* spikes, for *i* ≤ *l* are the times of occurrence of the *i*th spike in the response, measured relative to the start of the response window. For *i* > *l*, the *i*th spike is assumed to occur at a very large time delay. There are several reasonable choices for this value, but for simplicity we use *t*_*i*_ = ∞∀*i* > *l*. This results in vector elements $\frac{1}{t_{i}}=0\forall i\;>\;l$ (in particular, this means that the null response is represented as a length *L* vector of zeros).

The protocol required two steps. The first step produced an estimate of the most appropriate number of response classes to use for a given data set. The second step estimated the effect of disturbances to spike timing on the mutual information. We used the following procedure for the first step:

1. Select a region of the responses to consider (the response window).
2. Represent each response with a vector, using the *inverse spike time* representation.
3. Construct a distance matrix *D* containing elements *D*_*ij*_ equal to the Euclidean distance between the vector representations of responses *i* and *j*.
4. Use mean-distance hierarchical clustering of *D* to construct a binary clustering tree *T* over the set of responses.
5. Determine the maximum number of clusters *M*_*c*_ for which we can effectively calculate an unbiased information measure. The calculation of our measure *m*_*db*_, and the limits on *M*_*c*_ are detailed in the section on debiasing.
6. For all values of *N*_*clust*_ from 2 to *M*_*c*_, classify the responses using the partition of *T* that provides *N*_*clust*_ classes, and compute the corrected information measure *m*_*db*_ between the stimulus labels and this set of response classes.

The first step yielded a particular value of *N*_*clust*_ that maximizes *m*_*db*_, and an associated maximum measurable mutual information estimate *m*_*db*_. We used the value of *N*_*clust*_ in the second step, which followed the procedure:

1. Select a range of time-scale parameters *j*_*sd*_ to test. For every value of *j*_*sd*_:
2. Create a set of responses by adding noise values to each spike time in the true response set. These noise values are drawn from a Gaussian distribution with mean 0 and standard deviation *j*_*sd*_. Optionally, we may increase the size of the response set during this step, by creating *n*_*jit*_ different copies of each recorded response, modified with different, independent, noise values.
3. Window the responses, using the same window as in the first step. Since this occurs after adding some timing noise, we will not always include the same set of spikes in the analysis window.
4. Calculate the matrix *D* and information measure *m*_*db*_ as in the first step, using the value of *N*_*clust*_ determined in the first step.

The result of this procedure is a vector of samples *m*_*db*_(*j*_*sd*_), showing the change in the information measure *m*_*db*_ as the exact timing of the response spikes is lost. To compare the control case and the case of blocking inhibition, these values were calculated for each case, independently, except that the value of *N*_*clust*_ used in step 2 is the average of the values determined for the two cases in step 1 (these values are typically similar but not identical). In this method, we follow the temporal coding protocol established in Montemurro et al. (2007), but instead of decreasing the precision of timing responses, we use full resolution spike trains, modifying the precision by injecting continuous spike time jitter noise, as in Hatsopoulos et al. (2003) and Amarasingham et al. (2012).

#### 2.2.3. Debiasing

Bias in estimates of mutual information is a well known and heavily studied problem (Panzeri et al., 2007). Estimates of mutual information for small numbers of measurements in a large space are typically biased upwards. Typically, comparisons between two measures of mutual information suffer less from biases than absolute measures, because the bias in the two samples is often correlated. In our analysis, however, we wanted to compare measures of mutual information calculated for different values of the number of response classes, *N*_*clust*_, and the amount of temporal precision in the responses (represented inversely by the noise value *j*_*sd*_). These parameters change the size of the response space. Because the number of measurements remains the same, this changes the bias. Consequently the estimated value of mutual information increases with *N*_*clust*_, and decreases with *j*_*sd*_, not only for real data, but also for the random artificial responses, which are known to have a true mutual information of 0 (since they are generated independent of the stimuli). Any true effects of timing precision on the neural code are thus confounded by the effect of increasing the ratio of response space size to number of measurements.

A variety of debiasing strategies exist for mutual information measures. We tested several of these, included the Panzeri-Treves, Quadratic extrapolation, and Nemenmann-Shafee-Bialek estimators, as well as the shuffle corrections to these estimators, as implemented by *pyentropy* (Ince et al., 2009). Although these methods resulted in a downward shift of the estimates of mutual information for any given value of *N*_*clust*_ or *j*_*sd*_, they did not remove the trends observed for changes in these parameters, even in the random test data.

In order for a difference of mutual information estimates to reduce bias, we needed to consider estimates which use the same estimator, space sizes, sample sizes, and approximate distribution of samples. We tested for estimate bias by comparing estimates from physiological datasets, to estimates from responses generated by a homogeneous Poisson process with an equivalent spike rate. For our final estimator, we improved slightly on this comparison using a correction of the following form, which takes a parameter *n*_*reps*_:

- Estimate the mutual information *m*(*S, R*) between the set of stimulus identities *S* and response classes *R*, using the direct estimator.
- *n*_*reps*_ times:
  - Calculate a random permutation of the response list, *P*_*i*_.
  - Calculate the mutual information *m*(*S*, *P*_*i*_) using the direct estimator.
  - Calculate the difference *m*^*i*^_*db*_ = *m*(*S, R*) − *m*(*S*, *P*_*i*_).
- Return the mean and standard deviation of the set of {*m*^*i*^_*db*_}. The mean (*m*_*db*_) is used as the mutual information estimate, and the standard deviation as a confidence interval for this estimate.

The logic for this estimator is that the random permutation of the response should remove any true mutual information between the stimulus and response lists, but should have no effect on biases, which depend on the number of samples and the distribution of responses. Consequently, if *m*(*S, R*) is greater than the *m*(*S*, *P*_*i*_), then the difference (*m*^*i*^_*db*_) should reflect only true correspondence between the stimulus and response lists. This method is an improvement on subtracting an estimate of mutual information for Poisson data in that the distribution of shuffled response classes has the same entropy as the true distribution of response classes, where the distribution of classification of random responses might have a different entropy.

It is worth noting that the result of debiasing in this way has some properties that are not expected for mutual information. In particular, the mutual information between the stimulus set and any clustering of the response set should be monotonically increasing with the number of clusters retained. This property does not necessarily apply to *m*_*db*_, however. Particularly, as mentioned, for the values that we are considering for size of response space, distribution of responses, and sample size, it is common that the mutual information estimates for random responses reach the stimulus entropy (4 bits, in this case). This is a global upper bound for mutual information between this stimulus set and any response set. This value can be attained by randomly generated responses, but also by the random permutations *P*_*i*_. This effect can be viewed as a compression of the measurable range of mutual information. For a stimulus with entropy *e*_*s*_, the difference *e*_*s*_ − 〈*m*(*S*, *P*_*i*_)〉 is a measure of the mutual information which is meaningfully detectable by estimator *m*, given the limits of sample size. Although grouping responses can't increase the mutual information, for small data sets it will typically increase this *accessible* mutual information, by way of increasing the ratio of the number of measurements to the size of the response space. Consequently, the measure *m*_*db*_(*n*_*clust*_) typically has a maximum value that occurs at intermediate values between the limits of small *n*_*clust*_, where the true mutual information is lost through grouping, and large, where the accessible *n*_*clust*_ is lost through non-correctable biases.

**Figure 1:**
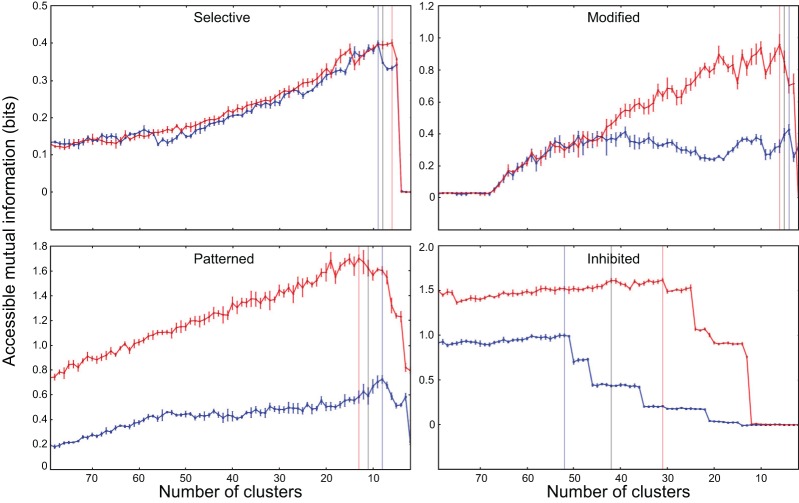
**Application of the clustering mutual information protocol to exemplars from the four neuron classes (see section 3)**. Number of clusters are shown on the horizontal axis (with high numbers, implying more information, shown on the left). *Accessible* mutual information is plotted on the vertical axis. Typical neurons were selected from classes (1) *selective*; (2) *modified*; (3) *patterned*; and (4) *inhibited*. This analysis returns two important measurements. The first is the peak value of accessible information for each neurons, marked by a vertical line for each experimental condition (control; blue and inhibition blocked; red). The second is the number of clusters used to calculate the accessible information. The measurements reported here were calculated on responses using a time window beginning 10 ms after stimulus onset and lasting 153 ms (ending 20 ms after the offset of the longest stimulus).

Figure 1 shows the application of the mutual information protocol to four different neurons. This analysis returned two important measurements. The first is the peak value of *accessible* information for the neuron, and the second is the number of clusters used to calculate this value. We used the peak value to classify neurons as non-responders (those neurons where the peak accessible mutual information was not significantly greater than zero), which we eliminated from subsequent analysis. We used the optimal number of clusters when calculating information in the subsequent analysis of the neurons.

For small enough values of the number of response clusters *n*_*clust*_, the debiased statistic *m*_*db*_ seems to accurately reflect true correspondence between stimulus and classified response, and is thus an appropriate measure for investigating the coding properties of the responses. Particularly, randomly generated artificial data and shuffled real data result in an *m*_*db*_ = 0, while artificial data that reflects actual coding of the stimulus, as well as real data from most of the observed cells, results in an *m*_*db*_ > 0.

#### 2.2.4. Rate corrections

In most cases, application of bicuculline and strychnine increased overall spike rates. This increase often resulted in an increase of total mutual information, and a decrease of information per spike. To determine how elements of the neural code such as temporal precision, response reliability, and stimulus selectivity were affected by blocking inhibition, we needed to separate the effects of changes in spike rates from these other effects, when comparing between the control and drug cases. This can be difficult when looking only at clusters or mutual information measurements, which, in the control case, are severely limited by the low spike rates.

One approach is to consider mutual information per spike, rather than total mutual information. In the majority of cases we found that removing inhibition increased the total mutual information, but decreased the information per spike (meaning that the increase in information is less than the increase in rate). This result is quite reliable, but often not very satisfying. Particularly, in a cell with a reasonably high mutual information in the control case, the relatively low (4 bit) entropy of our stimulus space will often provide an upper bound on the mutual information that ensures that increases in rate **cannot** provide proportional increases in mutual information, regardless of how well the additional spikes correspond to structures in the stimulus waveforms.

An additional approach we used in addressing the spike rate issue is founded on the hypothesis that the sparsely firing cells may operate as elements of a population of similar cells. In this case, during presentations when the cell that we are recording is silent, other similar cells may be active, which may provide input to the same downstream structures, and implement the same code. Within the limits of our data, we can approximate such a population code by using multiple responses from the same cell to different presentations of a stimulus. We construct a virtual response which is the superposition of several actual recorded responses. This requires data sets with larger numbers of presentations, but the result is that we can synthesize “population” responses for the control case and the drug case which contain about the same number of total spikes (by virtue of using more repetitions per response in the sparser control case). Nevertheless, in cases for which the response may consist of only one or two spikes, even the careful corrections we perform here may not be sufficient to offset the sparsity of the response, and the information timing methods may not detect precise timing even when it is present in those cases. All subsequent results should be interpreted with this limitation in mind.

Implementation of this method of correction is a pre-processing step. Before data are analyzed, we run a response combination function which assembles composite response. This function ensures that the expectation firing rates in the control and drug condition are approximately similar. Subsequent analyses and visualizations look the same as without the correction.

#### 2.2.5. Clustering

Mutual information measures are expected to reflect on how **well** the stimuli are coded by the responses, but do not provide any explicit statements about **how** they are coded. It is possible that blocking of inhibition might result in a significantly different coding of stimuli, but one that, by chance, contained a similar amount of information and depended on a similar level of timing precision. To address this possibility, we performed hierarchical clustering of the stimuli based on the response space. We then compared the resulting clustering trees.

This analysis used some similar techniques to the mutual information measurements, including construction of a distance matrix and mean-distance-based hierarchical clustering of that matrix. The fundamental difference is that in this case we explicitly used the stimulus-conditioned responses *R*(s) associated with a particular stimulus *s*. We thus constructed a distance matrix, *C*_*ij*_, such that element (*i, j*) is the mean value of the set of pairwise dis-similarities between elements of *R*(*s*_*i*_) (responses to stimulus i) and *R*(*s*_*j*_).

For this purpose, we found that there is a serious difficulty with using any true distance measure as the dis-similarity measure. The issue is that the expected magnitude of most distances between spike trains depends on the number of spikes. In particular, in sparsely firing cells such as those recorded in IC, two almost identical complex bursts should intuitively provide more information about the similarity of the stimuli evoking them than two empty responses because the latter are likely to occur in response to almost any possible stimulus. True distance functions, however, report the empty responses to be identical, and the long responses with mild differences in spike timings (or number) to be significantly distant. Indeed, under a distance function that includes high precision in the measurement of spike timing, empty responses are typically the *only* pairs of responses that have 0 distance. Due to the nature of hierarchical clustering, these groups of null responses end up being treated as highly informative and result in significant changes to the overall clustering. Blocking inhibition tended to increase spike rate and reduce the number of null responses and this can have a significant, but rather trivial, effect on clustering under these distances.

Our solution was to use a normalized dis-similarity measure. We used a measure based on the distance *D*^*spike*^_*q*_, described by Victor and Purpura (1997) (which we refer to as the *Victor distance*). Our variant, the *Victor distance per spike* (*vdps*), was calculated as follows: $vdps_{q}(s_{1}s_{2})=\frac{D_{q}^{spike}(s_{1}s_{2})}{\left\|s_{1}\|+\|s_{2}\right\|}$where ‖*s*_*i*_‖ denotes the number of spikes in response *s*_*i*_. Additionally, for ∅ indicating a response containing no spikes, *vdps*_*q*_(∅, ∅)≡1 ∀ q.

In Victor's formulation of the *D*_*q*_ family of metrics, the parameter *q* represents the cost of transformations which move a spike in time, and is in units of, e.g., *seconds*^−1^ or *sample*^−1^. The traditional choice for *q* in our case would therefore be μ*s*^−1^. We chose to represent *q* in terms of a characteristic time-scale of the responses. This value is the longest time of separation, in samples, under which the *D*_*q*_ algorithm will still choose to represent two events as the same spike. This value (*q*) is related to the traditional *q*_μ*s*^−1^_ by $q=\frac{2}{q_{\mu s^{-1}}}$.

Note that (unlike *D*^*spike*^_*q*_) our measure *vdps* is, by design, not a true metric, since (a) *vdps*_*q*_(∅, ∅) ≠ 0, even though ∅ = ∅, and (b) *vdps*_*q*_ does not always obey the triangle inequality. Despite the failure of *vdps*_*q*_ to operate as a true metric, in practice it is superior to *D*^*spike*^_*q*_ as a grounds for clustering responses, particularly in sparsely firing systems where empty responses are common. Additionally, since *vdps*_*q*_ explicitly depends on the time-scale parameter *q*, it is straightforward to determine the effect of timing precision on clustering by computing the results over a range of *q*.

Using these dis-similarity matrices, we constructed clustering trees showing the similarity of stimuli. We constructed cluster trees using the *linkage* command in Matlab. To establish consistency in trees given the uncertainty of electrophysiological responses, we constructed repeated clusters after adding small amounts of noise to the dis-similarity matrices. We then took the majority rule consensus tree (Felsenstein, 1985) over these re-sampled trees as the representation of the sensory coding function for each neuron. Once tree representations were prepared for the different experimental conditions, we assessed whether there were any differences in coding properties by using the graph edit distance between trees (Zhang and Shasha, 1989). A small edit distance (a few nodes) indicates essentially equivalent representations. A large edit distance means a significant change in coding properties between pairs of experimental conditions. If such a change was detected, we examined the trees and isolated the nodes leading to the change in order to characterize the majors sources of representation difference.

#### 2.2.6. Artificial test systems

To assess the performance of the various methods, we considered test data generated by several artificial models. Each of these models was implemented as a function that could be called with a desired response length, spike rate, a characteristic time scale (precision), and a stimulus identity (integer), and would return a pseudo-random response spike train. The models used were:

*hPois* A homogeneous Poisson process, which generated responses not correlated to stimulus identity (this model ignores the precision and stimulus inputs).
 *rate* A rate coder, which generated responses that were individually homogeneous Poisson, but in which the rate was determined by stimulus identity (this model ignores the precision input).
  *tgc* A model that produces spikes in two groups, with the spacing between the groups dependent on stimulus identity and the precision parameter. The groups may contain random numbers of spikes with noisy positions, determined by internal parameters of the model. The total number of spikes is, on average, not dependent on the stimulus.

We expected that a successful analysis method would show no stimulus/response mutual information in the case of responses from *hpois*, would show mutual information, but no dependence of the information on spike timing for *rate*, and would show information for responses from *tgc*, and would show this information falling off to zero as spike timing information was lost.

**Figure 2:**
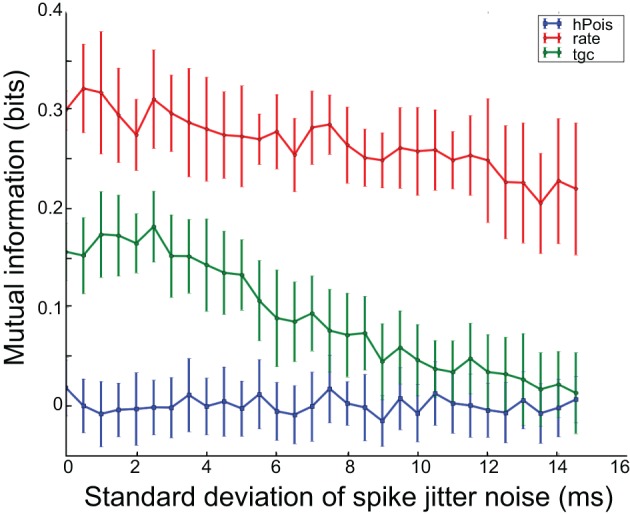
**Temporal precision of test systems**. Decreases in mutual information with increases in spike jitter indicate that information is lost when spike times are perturbed. Thus, a relatively constant function indicates little sensitivity to spike times such as with the rate code, whereas the rapidly decreasing function for the tgc system indicates high sensitivity to spike times, and hence a timing code.

Figure 2 shows the result of this measurement for the artificial data. The result is largely as expected. The *hPois* system has no mutual information at any noise value. The *tgc* system has information for small jitter values, but zero information for large noise values. This was expected because its code depends on spike timing. The true characteristic time scale of the *tgc* model is 6 ms, and this is accurately reflected by the fact that information about this model is retained for noise values smaller than this. The *rate* system shows information which is maintained up to large noise values, which is expected since its code does not depend on spike timing.

## 3. Results

We examined the effects of blocking inhibition on responses to vocalizations in 26 IC neurons. In nearly all neurons, blocking inhibition increased the spike rate evoked by the vocalizations. Across all neurons and stimuli, the average number of spikes per stimulus (200 ms window following stimulus onset) was 1.8 in the control condition (min 0.1, max 8.5, std 2.1), and increased to 4.8 after the application of bicuculline and strychnine (min 0.2, max 12.6, std 3.8). With one exception, all neurons had negligible spontaneous firing rate under both conditions. As we have documented previously (Mayko et al., 2012), blocking inhibition decreased the selectivity of neurons in the IC, with an increase in the number of stimuli that evoked responses.

Four of the neurons (15%), although they fired spikes, did not appear to respond to the vocalization stimuli we presented (the maximum value of the debiased information estimate *m*_*db*_ was near 0). These neurons were not analyzed any further. The average number of stimuli that evoked at least one spike from the 22 responsive neurons increased from 7.5 under control conditions (min 1, max 12, std 3.5) to 9.1 with inhibition blocked (min 1 max 13, std 3.1).

**Figure 3:**
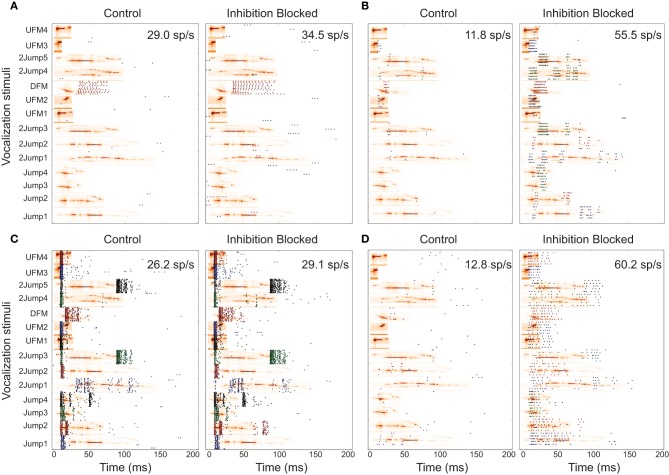
**Neural responses from different classes**. Panel pairs contain responses in control condition and inhibition blocked condition, respectively. Responses are shown for vocalization stimuli, at the preferred attenuation. Responses are represented as a spike raster, with each detected action potential indicated by a dot. The horizontal axis represents time of occurrence with 0 being the start of stimulus presentation. Responses to the 14 different vocalizations are represented along the vertical axis and vocalization names are indicated. These vocalizations are types of commonly emitted syllables (Mahrt et al., 2013). The background images show the spectrograms of each vocalization. Each panel contains the average response spike rate, computed as *total number of spikes/number of trials eliciting response/response window* (158 ms here). **(A)** *selective* neuron; **(B)** *modified* neuron; **(C)** *patterned* neuron; **(D)** *inhibited* neuron.

We observed a variety of response patterns across the responsive 22 IC neurons. We classified these responses into four general classes, based on their response to stimuli in both conditions. Nine neurons (35%) responded strongly to one vocalization, with little to no response to any other (*selective*). Three neurons (12%) responded with highly consistent and distinguishable patterns to the majority of vocalizations, under both experimental conditions (*patterned*). Both of these classes had only minute changes when inhibition was blocked. Six cells (23%) gave selective response patterns to various stimuli. When inhibition was blocked, they responded more vigorously, and to a larger number of vocalizations (*modified*). Four neurons (15%) only responded when inhibition was blocked (*inhibited*). These responded with similar patterns to the *modified* group when inhibition was blocked, and are probably of the same class, for which our relatively limited stimulus set did not contain any of their preferred stimuli. However, due to the lack of any data in the control condition, they were omitted from most analyses comparing the conditions. Figure 3 shows response rasters from typical examples of the responsive cell classes. This classification is approximate and used mostly for convenience, in order to provide convenient labels for communicating general statements about groups of neurons with somewhat similar properties. For that reason, we list and summarize the classes here, rather than after presenting all the evidence for their properties.

### 3.1. Mutual information clustering

We evaluated the degree to which the response structure reflected stimulus identity using mutual information. Our objective was to determine whether neurons in IC represent vocalizations with distinguishable spiking patterns and whether inhibition in the IC plays a role in generating these patterns. In particular, we examined whether the representation of particular vocalizations depended on the precision of spike timing. In applying mutual information metrics to this investigation we faced a particularly difficult debaising problem (section 2.2). To address this issue, we calibrated several mutual information calculations and debiaising strategies against artificial data, where we knew the true structure of the stimulus representation (see Figure 2).

After categorizing the neurons into the four response types, we calculated the peak mutual information for each group under both the control and blocking inhibition conditions (Table 1). In addition to the raw mutual information, we calculated the information per spike. In some cases, blocking of inhibition did not change the mutual information, and in others it increased information, but in nearly all cases, blocking inhibition **decreased** information per spike. As information per spike is proportional to information per unit expended energy (assuming fixed metabolic cost of spikes), this seems to indicate one major function of inhibition; to decrease the overall cost of information transmitted out of the IC. The principle exception from this trend seems to be the *patterned* neurons, for which the information measures did not change significantly.

**Table 1:** **Information capacity of the various neuron classes in control and inhibition-blocked conditions**.

	**Control**		**Inhibition blockers**	
**Cell class**	**Max MI**	**Max MI/spike**	**Max MI**	**Max MI/spike**
*Selective*	0.57	3.32	0.50	1.38
*Modified*	0.85	1.68	1.77	0.39
*Patterned*	1.98	0.55	2.21	0.58
*Inhibited*	1.17	5.61	1.61	0.14

*max MI is the peak mutual information for each class (in bits). max MI/spike is the maximal class mutual information per spike (in bits/spike). Blocking inhibition increased the mutual information (except in the selective cells) however, it significantly decreased the information per spike*.

### 3.2. Temporal precision

We assessed the dependence of the stimulus representation on exact spike timing by tracking the changes in mutual information as we perturbed the spike timing with noise. We repeatedly estimated mutual information after altering the spike timing by adding noise values to each spike. Noise values were drawn independently from a Gaussian distribution with mean 0 and standard deviation which we varied systematically. We tracked the mutual information as a function of the standard deviation of the noise distribution, *j*_*sd*_. Noise values were added before windowing the responses, so it was possible for noise to move spikes into, or out of, the analysis window. Repeated measurements at the same noise level reflect randomness both in the noise samples, and in the shuffling order chosen during debiasing. Measurements are reported with mean values and standard deviations over at least 25 repeats (five debiasing choices each, for five noise choices).

**Figure 4:**
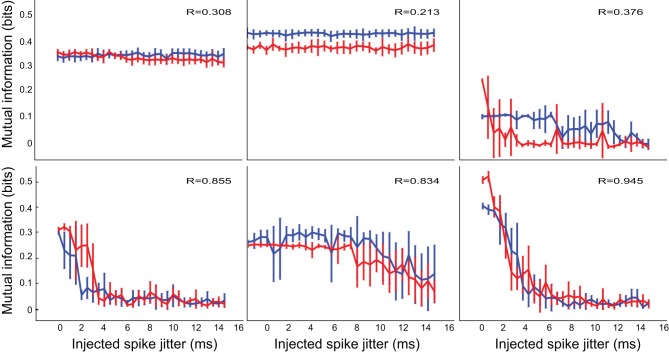
**The temporal precision of *selective* IC neurons was varied and not affected by inhibition**. The two experimental conditions are indicated in blue (control) and red (inhibition blocked). As with the test systems, constant functions indicate little sensitivity to spike timing, while rapidly decreasing functions indicate high sensitivity to spike times. The neurons show a spectrum of temporal precision, from rate coders (first two plots), to a highly precise and sensitive temporal coder (last plot). Here and on subsequent figures the value of the Pearson correlation coefficient *R* indicates the correlation between the control condition and the inhibition blocked condition, when one is considered a function of the other, with each jitter condition providing a point pair for the estimate.

When applied to electrophysiological data, the timing analysis revealed a broad spectrum of temporal precision, even for neurons within the same response class. *Selective* neurons (Figure 4) could perform their function with either a rate code (flat functions in the first two panels) or a range of temporal codes with different precision. In these neurons, removal of inhibition (red lines) did not seem to affect either the information processing aspects, or the timing aspects of the neurons. *Inhibited* neurons showed similar response patterns.

**Figure 5:**
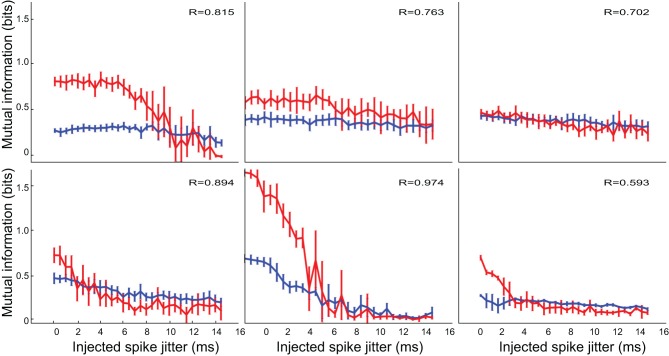
**The temporal precision of *modified* neurons was variable and blocking inhibition increased the amount of information**. The two experimental conditions are indicated in blue (control) and red (inhibition blocked).

**Figure 6:**
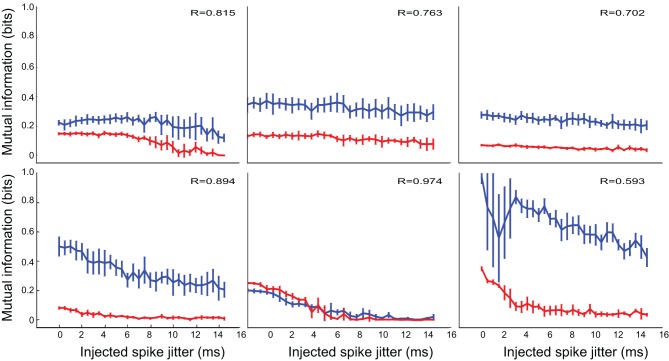
**Temporal precision for *modified* neurons, as measured by the mutual information in *bits per spike* (bps) of perturbed spike trains, exhibited the same trend**. However, in contrast to the situation in Figure 5, this information measure was higher in the control condition (blue trace) than with inhibition blocked (red trace): blocking inhibition decreased the amount of information *per spike*.

*Modified* neurons (Figure 5) exhibited a similar variety in temporal sensitivity, but these neurons displayed differences in their informativeness under the control and inhibition-blocked conditions. For all but one neuron (third panel in Figure 5), blocking inhibition increased the amount of information represented [control (blue) traces are above the inhibition-blocked (red) traces]. However, this increased informativeness comes at a high cost. If we consider the information per spike (Figure 6), we see that it is much higher in the control condition (blue trace) compared to with inhibition blocked (red trace). Because information per spike is proportional to information per unit expended energy, we interpret this result to reflect an energy optimization role of inhibition: single neuron capacity is somewhat decreased, leading to a comparatively large savings in energy of information representation.

**Figure 7:**
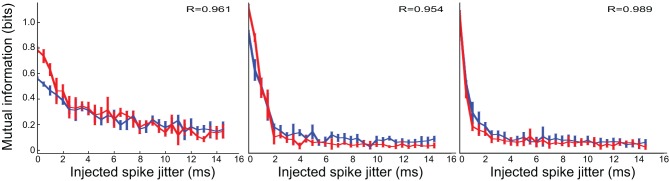
***Patterned* neurons display high temporal precision and this is not affected by inhibition**. The two experimental conditions are indicated in blue (control) and red (inhibition blocked).

*Patterned* neurons, shown in Figure 7 were different from the previous two groups in both aspects. First, these neurons universally demonstrated high temporal precision, often less than 2 ms. Furthermore, their information capacity was almost completely unaffected by removal of inhibition, both in the raw information measure, and in information per spike.

In the majority of neurons, information content and change of information with added noise were not dramatically influenced by inhibition. In some cases, where the absolute information was different between conditions, the relationship between information and timing noise was still very similar. We measured this similarity using the Pearson's *R* correlation measure between the two trajectories, which is reported on each panel. In cases where there was a significant dependence of information on timing precision, only one neuron shows a correlation with *R*<0.8 (bottom right panel on Figures 5, 6). That specific case may have been affected by the relatively large uncertainty in estimating the information measure (notice the large error bars on the blue trace on that panel). Of all neurons, only one (4.5% of the cases) showed any change in temporal precision, specifically, increased temporal precision under the treatment condition (upper right panel on Figure 4, red trace on that panel showing a marked decrease with jitter, compared to the relatively constant blue trace, and a correlation coefficient between the two *R* = 0.376, indicating distinct properties).

### 3.3. Stimulus representation

Mutual information measures are expected to reflect on how **well** the stimuli are coded by the responses, but do not provide explicit statements about **how** they are coded. It is possible (albeit unlikely) that blocking of inhibition might result in a significantly different representation of stimuli, but one that, by chance, contained a similar amount of information, and depended on a similar level of timing precision. As a first step in addressing this possibility, we analyzed the specific coding aspect of our IC neurons.

For *selective* neurons, the only comparative observation we can make is that they retain their selectivity. For neurons with responses to a range of vocalizations, the *patterned* and *modified* neurons, we can also ask about the relationship between representations of different vocalizations. For example, in those classes, two stimuli might generate responses that are distinguishable, but highly similar, and this could be relevant to downstream neurons. Here, we ask whether inhibition might shape these response similarities.

**Figure 8:**
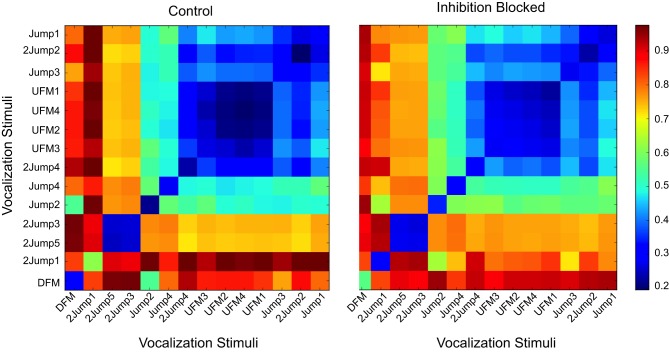
**Dissimilarity matrices for a broadly responsive neuron under control (left panel) and inhibition-blocked (right panel) conditions**. An element in this matrix, *D*_*i,j*_, contains the average dissimilarity between neural responses to pattern *i* and the corresponding responses to patter *j*, as measured by the *vdps* measure. This broadly responsive *pattern* neuron could discriminate rough classes of vocalization stimuli (blue regions, small dissimilarities), with high dissimilarity between them (red regions, off-diagonal regions). Stimuli with small cross-stimulus dissimilarity (large blue region in the top right quarter of the matrix, blue square for stimuli 2Jump5 and 2Jump3) are not distinguishable based on the spiking patterns of this neuron, but are highly distinguishable from other stimuli. There are a few specific stimuli that are discriminable [e.g., (DFM,DFM), a blue square with mostly red along across other stimuli]. In this neuron, blocking inhibition did not modify the neurons ability to discriminate among vocalizations.

For that purpose we constructed a response dis-similarity matrix that included information about stimulus identities. In this case, the matrix *D* of size (14,14) was determined by the number of stimuli, with *D*_*i*,*j*_ containing the average of pairwise dis-similarities between responses to stimulus *i* and responses to stimulus *j*. This produced a symmetric matrix of non-negative values. These matrices provide a visualization, and quantitative measure, of groups of stimuli that are related in the structure of their generated responses, as shown in Figure 8. The diagonal elements in these matrices are in general not zero. In fact, these elements are meaningful. They reflect the reproducibility of responses to the same stimulus. Off-diagonal elements of similar intensity indicate stimuli that are in general indistinguishable—the response-based dissimilarity between each other is low, comparable to the self-dissimilartiry of diagonal elements. Distinguishable groups have large dissimilarity between their corresponding elements (for example, the orange bands between the group (2Jump3, 2Jump5) and the large blue region to the left of UFM3). In that figure, a *patterned* neuron again demonstrates little effect when inhibition is removed: the pre- and post-treatment dissimilarity matrices are essentially identical.

**Figure 9:**
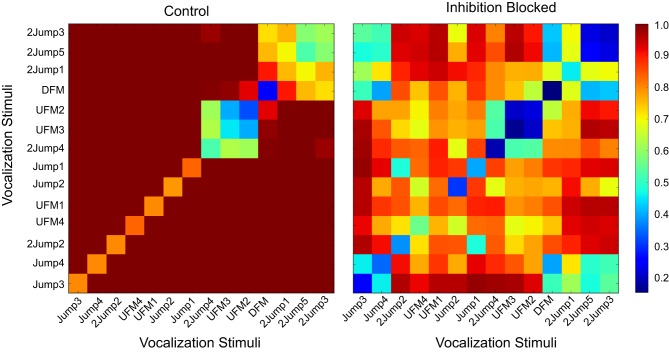
**Dissimilarity matrices for a *modified* neuron under control (left panel) and inhibition-blocked (right panel) conditions**. The response patterns of the neuron broaden with the blocking of inhibition, and become in general less precise (color in off-diagonal elements moved to yellow and green, indicating smaller distances). Inhibition decreases this neuron's selectivity and its ability to discriminate among vocalizations.

A different situation was observed for the *modified* neurons. The example neuron we show in Figure 9 was relatively selective in that it only responded to a few vocalizations (left panel). In particular, it had a very specific response to stimulus DFM (blue diagonal element, with orange and red off-diagonal terms). It also had somewhat broader responses to the classes (2Jump4,UFM2,3) and (2Jump3,5) (bluish-green block-diagonal groups, with red complementary terms). Blocking inhibition broadened these responses significantly, and made the responses to all other stimuli more similar, and hence less discriminable (right panel), as indicated by the general move toward yellow of the off-diagonal term. However, additional finer coding analysis for neurons like that, like the case shown later in Figure 11, indicated only minor representational changes when only *consistent* response classes were considered (defined below).

**Figure 10:**
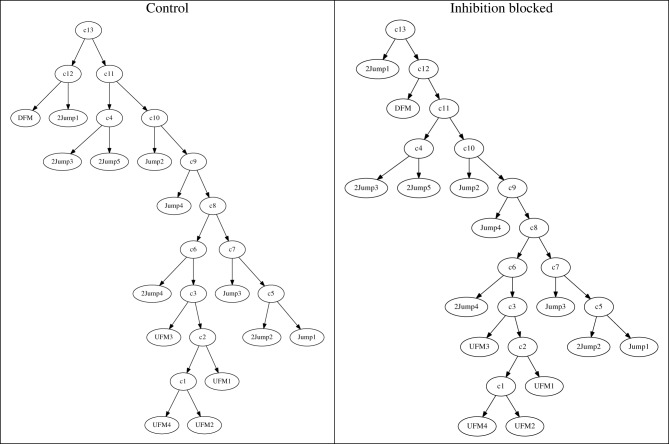
**Consistent consensus trees under control (left panel) and inhibition-blocked (right panel) conditions, for the neuron shown in Figure 8**. The two clustering trees are essentially identical. The only difference in this case is a switch of stimulus 30kHz2Harm from node *c12* to the root *c13*. This indicates that inhibition does not alter the neuron's representation of vocalizations.

Using these dis-similarity matrices, we constructed hierarchical clustering trees showing the grouping of stimuli as decoded through the similarity of neural responses. In the case of the *patterned* neurons, the results of this clustering are easy to interpret, in that it changes only minimally when inhibition is blocked. To establish that this effect is robust, we constructed repeated clusters after adding small amounts of noise to the matrices. We then took the majority rule consensus tree over these repeats. In *patterned* neurons, repeatability was high, consensus trees resemble individual trees nearly exactly, and the resulting consensus trees for the two experimental conditions were nearly identical (Figure 10). Specifically, in this case the only difference is that near the top, node *c12* groups 2 responses under the control condition (left panel), while under the treatment condition, one of those responses was attached to the tree root *c13* instead of node *c12*. Consensus trees for other cells exhibited similar minor differences (data not shown).

**Figure 11:**
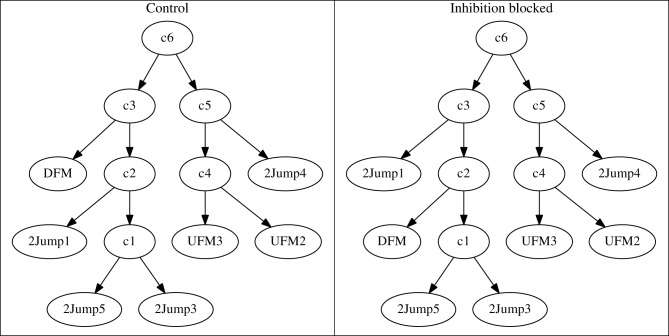
**Truncated response tree of *modified* neuron from Figure 9**. While the whole clustering tree (not shown) was relatively inconsistent, the truncated tree containing only the most robust classes showed consistent responses to specific classes of stimuli.

For the *modified* neurons, like the exemplar in Figure 11, interpreting the clusters was more difficult. Typically, in the control condition, a large number of stimuli resulted in no, or very few, responses. Grouping of these stimuli was therefore quite arbitrary and not robust to noise. The result was a consensus tree that only specified groupings among a small number of vocalization stimuli. When inhibition was blocked, a larger number of stimuli were typically grouped reliably. This resulted in clusterings that initially appear quite different, consistent with the apparently different dissimilarity matrices in Figure 9. However, if we consider only the subset of stimuli that were **reliably** grouped in both conditions (Figure 11), the clusterings were typically very similar. We call these stimuli (different ones for different neurons) *consistent* stimuli. In this case, the only change was the interchange of two stimuli, DFM and 2Jump1, between the neighboring nodes *c2* and *c3*.

Across all neurons of this type, the graph edit distances between clusterings generated by the same stimulus in different conditions were typically smaller than between random groupings, or groupings generated by a different neuron, but were typically larger than groupings generated by repeated measures within the same neuron and condition.

## 4. Discussion

In this study we examined whether inhibition in the IC plays a role in generating a timing code that may be used to discriminate among different vocalizations. We found that, in general, the balance between excitation and inhibition in the IC regulates the excitability and selectivity of individual neurons to vocalizations, but that inhibition does not play a major role in generating the temporal firing patterns to vocalizations. We also found that neurons in the IC use a variety of coding strategies to represent complex acoustic signals. On the selectivity spectrum, neurons ranged from very selective to broadly responsive (yet highly informative). Along the timing spectrum, neurons ranged from essentially rate coders, through mild use of spike timing information, to very precise neurons that were sensitive to spike timing on a millisecond time scale. Thus, the IC contains a highly diverse and complex representation of vocalizations that results in a variety of mechanisms for discriminating and possibly categorizing vocalizations.

### 4.1. Inhibition in IC affects response rates and selectivity to vocalizations but not temporal spike patterns

The most common (and expected), effect of blocking GABA_A_R and GlyR in the IC of awake mice was an increase in response rate. There are a number of potential microcircuits that could explain this effect. For example, the excitatory and inhibitory inputs could be co-tuned in frequency such that the inhibitory inputs decrease the response rate within the same frequency range of the excitatory inputs (Kelly and Caspary, 2005; Mayko et al., 2012) or the frequency tuning of the inhibitory inputs could be more broadly tuned than the excitatory inputs creating lateral inhibition (Yang et al., 1992; LeBeau et al., 2001; Mayko et al., 2012). Both of these microcircuits are thought to affect responses in the IC of mice to both simple stimuli and vocalizations (Mayko et al., 2012).

Inhibition has also been shown to play a role in creating selectivity to social vocalizations in the IC of mice and bats (Klug et al., 2002; Xie et al., 2005; Mayko et al., 2012). We have previously shown, using the same vocalization stimuli as in the current study, that altering the balance between excitation and inhibition by pharmacologically blocking GABAergic and glycinergic receptors in the IC decreases selectivity to social vocalizations in awake mice (Mayko et al., 2012), and we found the same results in the current study. Some of the observed selectivity loss may be due to the complementary action of bicuculine on calcium-dependent potassium channels. Inhibition may shape selectivity to vocalizations by keeping a neuron's membrane potential at subthreshold levels for some vocalizations and not others or by sharpening the excitatory frequency tuning curve so that fewer vocalizations contain energy that falls within the excitatory region (Portfors, 2004; Mayko et al., 2012).

Previous studies examining selectivity to vocalizations in the IC only used response rate as a metric for examining the influence of inhibition on encoding of vocalizations. Yet it is clear that at least some neurons in IC can use temporal coding for discriminating vocalizations (Schneider and Woolley, 2010; Woolley and Portfors, 2013). As far as we know, ours is the first study to examine the role of inhibition in shaping the fine temporal structure of neuronal responses to vocalizations in the IC. Our results clearly indicate that inhibition does not have any major effect on the temporal coding properties of IC neurons. When we compared the temporal precision of all IC neurons, blocking inhibition did not significantly modify the temporal dynamics of any of them (*R*>0.8 between the two conditions for neurons *that exhibited temporal coding*), except for a single neuron (4.5% of our population). This finding was somewhat unexpected as inhibitory inputs to IC neurons can influence latency (Park and Pollak, 1993; Le Beau et al., 1996) as well as shaping onset firing patterns (Le Beau et al., 1996; Jen and Zhang, 1999; Wu et al., 2006), and in light of the effects of bicuculline on fast potassium channels (Kurt et al., 2006). See Isaacson and Scanziani (2011) for a comprehensive review of the role of inhibition in shaping cortical responses.

The main effect of inhibition that we could detect was a marked *decrease* of information per spike in most cells in the *modified* class. Since this measure is typically a proxy for the energy efficiency of a neural code (Levy and Baxter, 1996), we interpret this result to reflect an energy optimization role of inhibition: inhibition decreases single neuronal capacity, while leading to a comparatively large savings in energy of information representation. The situation is compatible with the analysis performed in multiple other systems (Levy and Baxter, 1996; Laughlin et al., 1998; Laughlin, 2001; Lennie, 2003), indicating that high spike rates decrease the energy efficiency of a neural code, and a distributed neural representation is more energy efficient.

The neurons in the *patterned* class showed very small effects of blocking inhibition. Apart from a small rise in spike rate, inhibition does not seem to influence these neurons at all. Specifically, their coding properties seem completely invariant to the strength of inhibitory input. The robustness of coding observed in the unaffected neurons lead us to believe that they represent ethologically relevant information, which needs to be transmitted quickly (high timing sensitivity), regardless of metabolic cost.

We propose an *iceberg* model (Creutzfeldt et al., 1974; Rose and Blakemore, 1974; Isaacson and Scanziani, 2011) to explain our finding that inhibition in the IC does not affect the temporal spiking patterns of neurons to vocalizations. Under this model, the fine temporal structure of the response of a given neuron to a given stimulus is primarily driven by excitation, and inhibition is applied in a largely constant manner, which can be modeled as a change in the threshold at which the underlying excitatory structure is expressed in spikes. If this model is accurate, we expect that blocking inhibition would cause a previously hidden response structure to become more visible, as more of it exceeded threshold. This is in contrast to models where the excitation and inhibition interact on a fine time scale to shape the responses, such that blocking inhibition might be expected to, for example, change the occurrence times of the strongest components of the response.

### 4.2. Heterogeneity of timing codes in the IC

Considering that vocalizations vary in frequency content and amplitude over time, it is not surprising that neural responses to different vocalizations have different magnitudes and temporal firing patterns. Thus, response rate and/or temporal firing patterns may be used by individual neurons to different extents to represent vocalizations. Indeed, in our sample of IC neurons, some were essentially rate coders while others were sensitive to spike timing on a millisecond time scale. The most obvious temporal coders were the neurons in the *patterned* class with all of these using a timing code. However, in all other classes, a sizable subset of neurons exhibited high timing precision as well. Thus, we establish spike timing as an important feature for discriminating vocalizations in the mammalian IC. Similarly, in the MLd of zebra finches, individual neurons respond to different songs with different temporal spike patterns and many neurons discriminate songs using a timing code rather than a rate code (Schneider and Woolley, 2010). Very similar timing codes occur in the forebrain of birds (Wang et al., 2007) and the auditory cortex of guinea pigs (Huetz et al., 2011; Gaucher et al., 2013). Moreover, timing in the population activity of neurons in the human auditory cortex can be used to accurately identify segments of speech (Mesgarani and Chang, 2012; Pasley et al., 2012).

Given the much higher information capacity and energy efficiency of spike timing codes, one may wonder why a sensory system may be using a rate code. We considered several alternative explanations. A rate code is much easier to decode by downstream neurons than a more complex temporal code, so it may be needed for low-capacity stimuli of high significance. Or, it may be complementing the information of a temporal code by communicating different aspects of the stimulus (typically at slower temporal dynamics) (Huxter et al., 2003). We do not believe the latter is the case, as our measures did not register any temporal structure in the putative rate code. However, this may be a consequence of the restrictions of the experimental manipulation; presenting just 14 of the multitude of ethologically relevant stimuli may have triggered the rate response of neurons without their corresponding temporal representation. Further studies are needed to discriminate between these two cases.

### 4.3. Encoding of vocalizations in the inferior colliculus and auditory cortex

An interesting parallel of this work was performed on the auditory cortex of guinea pig (Gaucher et al., 2013). The two studies are only partially comparable because of differences in experimental manipulations. While both studies are concerned with the effects of inhibition on the corresponding auditory structures, in Gaucher et al. (2013) cortical inhibition was suppressed broadly through a topical application of GABA_A_ antagonists, while here we applied the antagonists more locally and directly onto the neurons we recorded from by using piggy-back electrodes (Havey and Caspary, 1980). Thus, we can address more specific questions about the effects of inhibition on individual neurons. However, for that we forgo the capacity of assessing the effects of inhibition on populations of neurons, which is one of the strengths of Gaucher et al. (2013).

In the cases where we can make a comparison, we also observed similar increases in firing rates in general and information rates for most of our neuron classes. In contrast, we did not observe the increase of spike timing reliability reported in Gaucher et al. (2013). This may indicate different processing strategies between the IC and auditory cortex, but it may also be a consequence of the different pharmacological protocols. Future experiments should more directly compare coding strategies for vocalizations along multiple sites in the auditory pathway.

## 5. Conclusions

There are multiple strategies by which an auditory system may represent information about stimuli. One way may be having highly selective neurons, where only one or two different vocalizations evoke a strong response from a single neuron. Another strategy is to have specific spike timing patterns for particular vocalizations such that each neural response can be matched to a specific vocalization. We found that both of these strategies were present in the IC. Furthermore, they were implemented with a varied degree of temporal precision, from rate codes on a time scale of 20 ms or larger (a long time scale for an ultrasonic system), to temporal codes on a sub-millisecond time scale. Thus, the IC employs diverse coding strategies for complex stimuli such as vocalizations.

Local inhibition played surprisingly little role in shaping the responses to vocalizations in the IC. When we disrupted the inhibitory inputs of individual neurons and thus altered the balance of excitation and inhibition, we found the expected rate increase in most neurons, but also found that the information content, stimulus representation and timing precision of the neural signals were essentially unchanged (and in some cases—completely unchanged). Thus, the major effect of inhibition in the IC seems to be a reduction of the overall spiking rate of the system, presumably to drive it to a more energy efficient regime.

### Conflict of interest statement

The authors declare that the research was conducted in the absence of any commercial or financial relationships that could be construed as a potential conflict of interest.

## Footnotes

^1^This is not always the highest amplitude. Some of the recorded cells responded more strongly to intermediate amplitudes.

## Supplementary material

Supplementary Table 1 **Individual cell classification and statistics under control and inhibition blocked conditions**. The reported statistics are: mean spike rate, mean response rate (spike rate to responsive stimuli), mutual information (MI) and MI per spike (MI/sp).
